# First Radiological Study of a Complete Dental Ontogeny Sequence of an Extinct Equid: Implications for Equidae Life History and Taphonomy

**DOI:** 10.1038/s41598-018-26817-3

**Published:** 2018-05-31

**Authors:** M. Soledad Domingo, Enrique Cantero, Isabel García-Real, Manuel J. Chamorro Sancho, David M. Martín Perea, M. Teresa Alberdi, Jorge Morales

**Affiliations:** 1 Department of Evolutionary Ecology, Doñana Biological Station-CSIC, Seville, Spain; 2 Department of Paleontology, Faculty of Geological Sciences, Complutense University of Madrid, Madrid, Spain; 3 Department of Paleobiology, National Museum of Natural Sciences-CSIC, Madrid, Spain; 4 Department of Animal Medicine and Surgery, Faculty of Veterinary Medicine, Complutense University of Madrid, Madrid, Spain; 5 Polyclinics Service. Military Centre of Veterinary. Ministry of Defence, Madrid, Spain

## Abstract

The sequence of cheek teeth mineralization, eruption, and replacement of an extinct horse species is here documented with radiological techniques for the first time thanks to the exceptional preservation of *Hipparion* sp. mandibles from Cerro de los Batallones (Madrid Basin, Spain). The sequence of dental ontogeny in mammals provides valuable insights about life history traits, such as the pace of growth, and about the mode of formation of fossiliferous assemblages. We have determined that the order of permanent cheek teeth mineralization and eruption of hipparionine horses is m1, m2, (p2, p3), p4, m3. Cheek teeth mineralization timing of hipparionine horses coincides with the one observed in modern equids. In turn, there are differences in the eruption timing of the p4 and m3 between horses belonging to the Anchitheriinae and Hipparionini compared to equids of the *Equus* genus that might be related to the shorter durability of the deciduous tooth dp4 in anchitheriine and hipparionine horses and, more broadly, to an increased durability of equid teeth through their evolutionary history. Based on the dental eruption sequence, hipparionine horses are slow-growing, long-living mammals. The *Hipparion* sp. assemblage from Batallones-10 conforms to an attritional model, as individuals more vulnerable to natural mortality predominate.

## Introduction

Horses constitute key taxa to investigate past terrestrial ecosystems because their fossils are very abundant in the Cenozoic fossil record, easily identifiable, and represent wide geographic and temporal ranges. The long, continuous, and intercontinentally widespread Equidae fossil sequence has long been used to prove, dispel, and analyse evolutionary patterns and processes^1–6^. Horse fossils, specially their teeth, have been the object of numerous paleoecological studies that try to infer past environmental conditions. These studies include stable isotope, micro and mesowear and hypsodonty analyses^7–11^. Besides, extinct horses play an important role as biochronological markers. For example, the entrance of hipparionine horses, the taxa under study in the present work, in Eurasia and Africa from North America constitutes a crucial biochronological event (=the *Hipparion* Datum) to recognize the start of the Upper Miocene in the terrestrial deposits from these continents^12,13^. Precisely, hipparionine horses constitute a group of great prominence in the evolutionary history of equids as they were very abundant and speciose during the Late Miocene and Pliocene of Eurasia, Africa and North America^3,6^.

The study of complete dental ontogenetic sequences can provide valuable information about the life history and evolution of past species. Specifically, the timing of the permanent dental eruption is related to the postnatal pace of growth in a broad number of taxa, as summarized by the Schultz’s Rule^14–18^. This rule advocates that a delayed eruption of molars compared to permanent premolars can be associated with a slow pace of growth^14,15^. The study of the dentition ontogeny also allows us to infer the age at death of the individuals from a fossil site and, by analysing the age-frequency distribution, the causes behind the death and the mode of formation of a taphocoenosis can be assessed^19–21^. In any event, the dental ontogeny sequences remain unknown for most extinct mammals. Only paleontological sites with exceptionally rich and well-preserved fossil assemblages provide an adequate number of specimens (including juvenile individuals) to describe ontogenetic series^16,19–29^. This is the case of Cerro de los Batallones fossil sites, which are known for containing diverse and extremely rich mammalian fossil assemblages^30,31^. Cerro de los Batallones is located in the Madrid Basin (Spain) and is composed of nine fossil localities whose fauna allowed us to assign a Late Miocene age for all of them^12,32,33^ (10–9 Ma; MN10 unit). Among the many species represented at the Cerro de los Batallones sites, *Hipparion* sp. (=*Hippotherium* sp.) from the site of Batallones-10 stands out as it is represented by one of the largest and best-preserved assemblages for the Hipparionini tribe not only in Spain but also in Europe and is constituted by a large sample of skulls and mandibles with their dentition in place. Remains from Batallones-10 belong to a single *Hipparion* species that is still under study.

As far as extinct horses are concerned, only a few studies have described their dental ontogeny, mainly based on visual inspection (no radiological techniques were used) of maxillary bones, mandibles or isolated teeth (Table 1). Recently, Li *et al*.^29^ used X-ray techniques to analyse the dental ontogeny of the late Miocene hipparionine horses from the Lamagou fauna of Fugu (China). Nevertheless, their X-ray sample belonged to adult horses exclusively, so they described wear patterns on permanent dentition but not the complete sequence of mineralization, eruption and replacement. At Cerro de los Batallones, *Hipparion* sp. infantile and juvenile individuals are very abundant and, therefore, the complete dental ontogenetic sequence can be described.

**Table 1:** Eruption sequence of permanent cheek teeth in living and extinct equids.

Species	Fossil site	Age	Eruption sequence of permanent cheek teeth	Crown height^a^	Taxonomic adscription	Mortality type	Reference
*Equus caballus*		Modern	*m1, m2, p2, p3, m3, p4*	Hypsodont	Equini (Equinae)		^40^
*Equus asinus*		Modern	**m1, m2, p2, p3, p4, m3**	Hypsodont	Equini (Equinae)		^39^
*Equus hemionus hemionus*		Modern	*m1, m2, (p2, p3, m3), p4*	Hypsodont	Equini (Equinae)		^63^
*Equus burchelli antiquorum*		Modern	*m1, m2, (p2, p3, m3), p4*	Hypsodont	Equini (Equinae)		^36^
*Equus burchelli*		Modern	**m1, m2, p2, p3, p4, m3**	Hypsodont	Equini (Equinae)		^64^
*Equus zebra zebra*		Modern	**m1, m2, p2, p3?, p4, m3**	Hypsodont	Equini (Equinae)		^65^
*Equus zebra hartmannae*		Modern	*m1, m2, (p2, p3, m3), p4*	Hypsodont	Equini (Equinae)		^66^
*Equus simplicidens*	Hagerman Horse Quarry (USA)	Late Pliocene	*m1, m2, (p2, p3), m3, p4*	Hypsodont	Equini (Equinae)	Catastrophic (but previously interpreted as attritional)	^51^
*Protohippus* cf*. P. perditus*	Verdigre Quarry (USA)	Early Pliocene	**m1, m2, (p2–p4)** ^b^ **, m3**	Hypsodont	Equini or Protohippini (Equinae)	Catastrophic	^19^
*Hipparion chiai* and *Hipparion* cf. *H. coelophyes*	Lamagou Fauna of Fugu (China)	Late Miocene	m1, m2, p2, p3, (p4, m3)	Hypsodont	Hipparionini (Equinae)	Not reported	^29^
*Hipparion* sp.	Batallones-10 (Spain)	Late Miocene	**m1, m2, (p2, p3), p4, m** **3**	Hypsodont	Hipparionini (Equinae)	Attritional	This study
*Merychippus primus*	Sheep Creek (USA)	Middle Miocene	**m1, m2, (p2, p3), p4, m3**	Mesodont	Primitive Equinae	Catastrophic	^22^
*Parahippus leonensis*	Thomas Farm (USA)	Early Miocene	**m1, m2, (p2–p4)** ^c^ **, m3**	Brachydont	Anchitheriinae	Attritional (but taphonomic underrepresentation of juvenile remains)	^24^
*Archaeohippus blackbergi*	Thomas Farm (USA)	Early Miocene	**m1, m2, (p2–p4)** ^c^ **, m3**	Brachydont	Anchitheriinae	Intermale combat	^26^
*Propalaeotherium hassiacum*	Geiseltal (Germany)	Middle Eocene	m1, m2, m3, (p2–p4)	Brachydont	Palaeotheriidae (Equoidea)	Not reported	^28^

Fossil species are listed from younger to older. The mortality type is provided for fossil sites. Sequences where the p4 erupts before the m3 are in bold. Sequences where the m3 erupts before the p4 are in italics. Parentheses enclose teeth with more or less simultaneous eruption timing. ^a^Crown height follows Mihlbachler *et al*.^10^. ^b^The order of eruption of premolars is not specified. ^c^The authors specify that the p4 erupts at the same time as the other premolars.

The aim of this study is to describe for the first time with radiological techniques the postcanine dentition ontogeny (mineralization, eruption, and replacement patterns) of hipparionine horses, a fundamental taxonomic group for the equid evolutionary scheme and a very common faunal element in Neogene sites worldwide. These sequences will be compared with those known for extinct and extant equid species in order to contribute information about their life histories, specifically about their postnatal pace of growth, paying special attention to changes that might have occurred through the evolutionary history of Equidae. Successive age classes will be described for the Batallones-10 *Hipparion* sp. sample and age-frequency distribution data will be analysed to make taphonomic inferences about the cause behind the death of this exceptional hipparionine horse assemblage.

## Results

We have defined seven age classes based on dental sequences of hemimandibles and complete mandibles of 28 *Hipparion* sp. individuals from Batallones-10 (see below and Supplementary Table S1). Together with the described ontogenetic stages, we provide the approximate age when the same dental events occur in modern equids. The absolute age of each dental ontogenetic stage for *Hipparion* sp. remains, nonetheless, uncertain. Studies dealing with the life history of Miocene hypsodont equids suggest that their longevity (11–15 years) was shorter than the longevity of modern equids (20–25 years) reaching maturity earlier than the modern *Equus* and, consequently, it has been suggested that the dental sequence of ontogenetic events happened earlier^3,19,22–24,34,35^. It is worth highlighting that even in modern *Equus*, there are differences in the ages at which dental events occur depending on the *Equus* species (Supplementary Table S2). The corresponding age of dental events for modern *Equus* species that we provide here is based on the works of Smuts^36^ for *Equus burchelli*, Levine^37^ and Dixon and Copeland^38^ for *Equus caballus* (pony), Misk and Seilem^39^ for *Equus asinus* and Hoppe *et al*.^40^ for *Equus caballus* (Supplementary Table S2).

**Figure 1:**
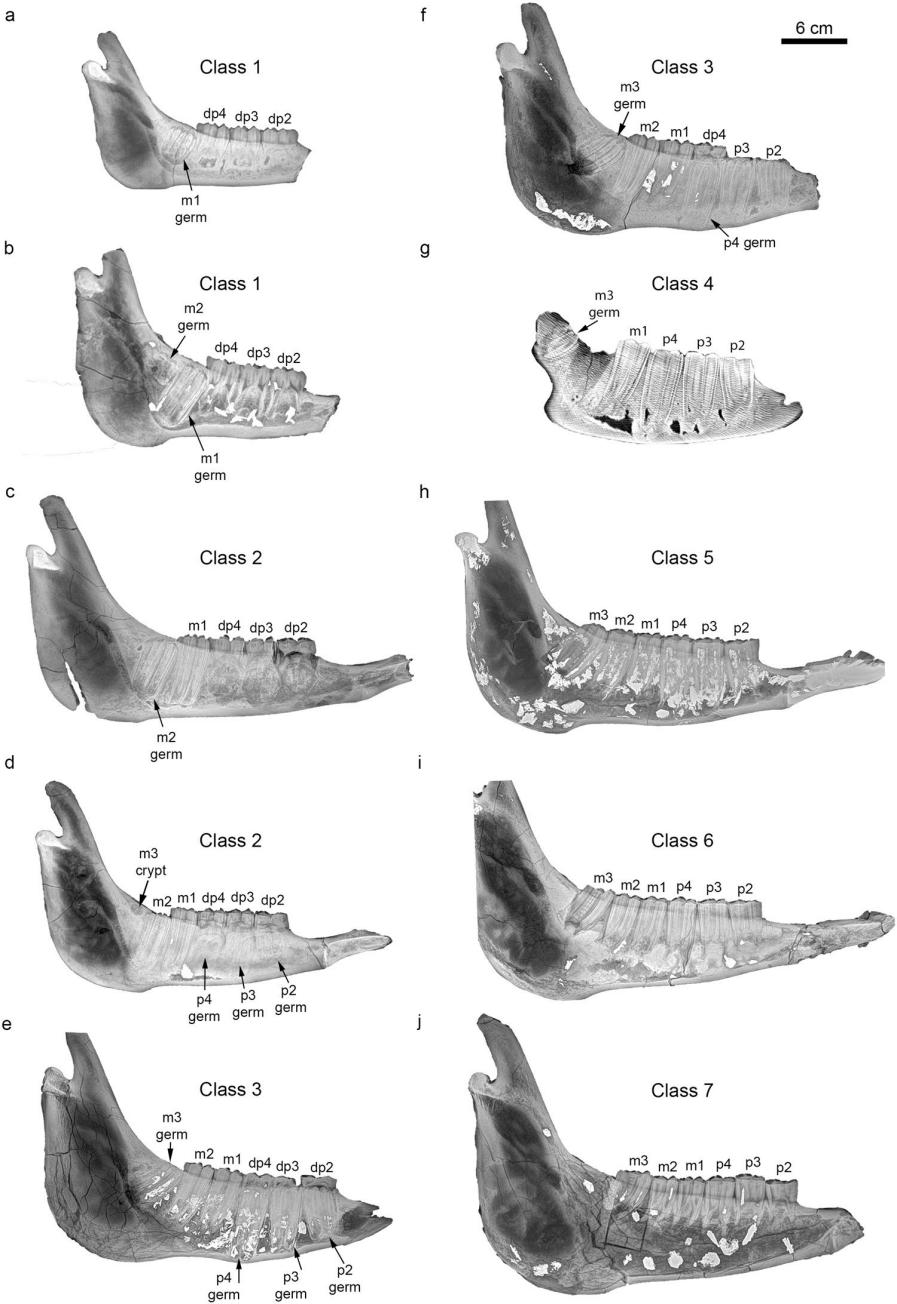
X-ray (**a, b, c, d, e, f, h, i, j**) and CT scan (**g**) images showing the sequence of cheek teeth mineralization, eruption and replacement of *Hipparion* sp. from Batallones-10. (**a**) Right hemimandible BAT-10′12 F5-17 (Age class 1). (**b**) Right hemimandible BAT-10′11 D6-56 (Age class 1). (**c**) Right hemimandible BAT-10′08 F4-47 (Age class 2). (**d**) Right hemimandible BAT-10′10 E3-26 (Age class 2). (**e**) Right hemimandible BAT-10′11 G2-135 (Age class 3). (**f**) Left hemimandible (mirrored) BAT-10′09 F3-76 (Age class 3). (**g**) Right hemimandible BAT-10′13 E3-42 (Age class 4). (**h**) Left hemimandible (mirrored) BAT-10′07 H3-154a (Age class 5). (**i**) Right hemimandible BAT-10′12 D6-139 (Age class 6). (**j**) Right hemimandible BAT-10′08 D5-25 (Age class 7).

The seven age classes described for *Hipparion* sp. from Batallones-10 are (Figs 1 and 2; Supplementary Table S1; Figs S1–S7):

- Class 1: dp2-dp4 are completely emerged and lightly worn. The germ of the m1 is visible within the jawbone and shows an increasing height of the crown as it mineralizes. The crypt for the m2 might be absent or present. Also, the germ of the m2 might be slightly visible in the most advanced stages of this age class (Fig. 1b). In modern equids, this age class would be given a minimum age of 1 month because deciduous premolars are a little worn. Since the m1 is close to eruption but not yet erupted in mandible BAT-10′11 D6-56 and the m2 is starting to mineralize (Fig. 1b), the maximum age for this age class would be around 7 months in modern equids. This age class is represented by 5 individuals.
- Class 2: dp2-dp4 are more worn than in Class 1. The germs of the p2 and p3 become visible in this age class. The same is true for the germ of the p4 although it becomes visible shortly after p2 and p3 are visible (Supplementary Fig. S2). The m1 is erupting or erupted and it reaches the full wear condition in the most advanced stages of this age class (Fig. 1c,d). In this age class, the m2 goes from the germ condition to the start of eruption (Fig. 1c,d). The crypt for the m3 might be absent or present. In modern equids, this class would have a minimum age of 8–10 months (the start of the m1 eruption) and a maximum age of 15–19 months (the start of the m2 eruption and the start of the p4 mineralization). This age class is represented by 10 individuals.
- Class 3: dp2-dp4 are more worn than in previous classes. Besides, the dp2 and dp3 might have just been shed and the p2 and p3 are beginning to erupt (Fig. 1f). The dp4 is still in place. The m1 is in wear. The m2 is almost in wear or in full wear. The m3 germ is visible below the mandible. In most modern horses, this age class would span from 18–24 months (the m2 is not yet in full wear) until 2.5–3.5 years (the p2 and p3 start to erupt). In the case of *Equus asinus*, Misk and Seilem^39^ propose that the p3 starts its eruption at around 27 months. This age class is represented by 4 individuals.
- Class 4: p2, p3, m1 and m2 are erupted and in wear. The p4 is erupted but has not reached full wear (Fig. 1g). The m3 is not erupted yet. It becomes more complicated to estimate the corresponding age for modern horses for this age class and the following ones since the works cited above mainly deal with modern horses where the m3 erupts before the p4, whereas in our *Hipparion* sp. sample the p4 erupts before the m3. Only *Equus asinus* from the study of Misk and Seilem^39^ shows the same pattern displayed by *Hipparion* sp. These authors indicate that the timing of the p4 eruption in *Equus asinus* is around 30 months, so this Class 4 would correspond to a horse slightly older than 30 months since the p4 is almost in wear. This age class is represented by 1 individual.
- Class 5: p2-m2 are erupted and in wear. The m3 is erupted but remains unworn or lightly worn (Fig. 1h). Following Misk and Seilem^39^, this age class would correspond to a modern horse slightly older than 3 years. This age class is represented by 2 individuals.
- Class 6: all permanent cheek teeth are erupted and in wear (Fig. 1i). This age class would correspond to modern horses older than 4 years. It is represented by 3 individuals.
- Class 7: all permanent cheek teeth are erupted and have lost much of their crown reserves. The p2 and m1 are close to the gum line (Fig. 1j). Based on the visual inspection of X-ray images shown by Dixon and Copeland^38^, we estimate that this age class would correspond to a modern horse that is older than 6 years. This age class is represented by 3 individuals.

**Figure 2:**
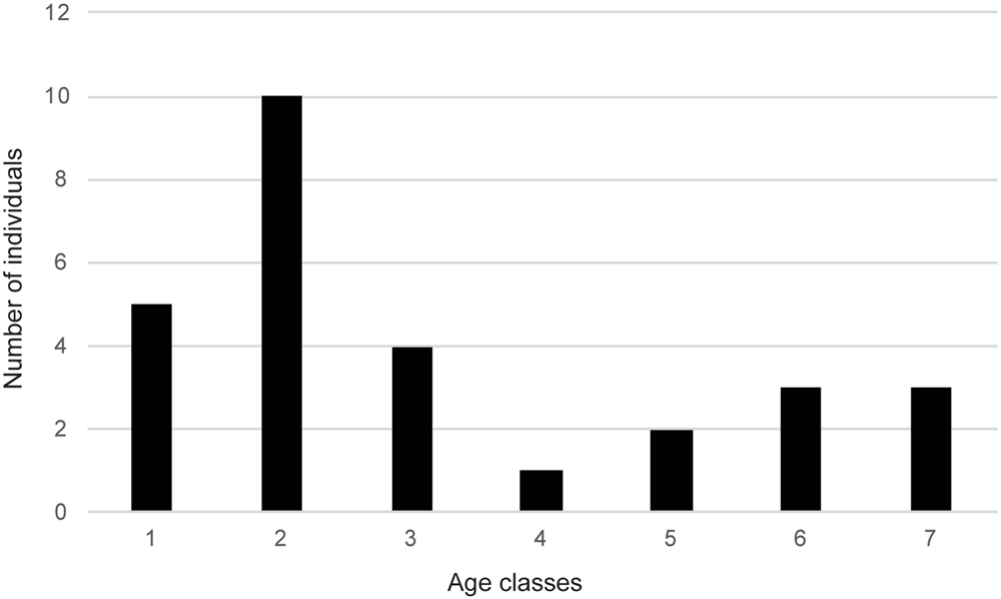
Mortality profile of *Hipparion* sp. from Batallones-10.

**Figure 3:**
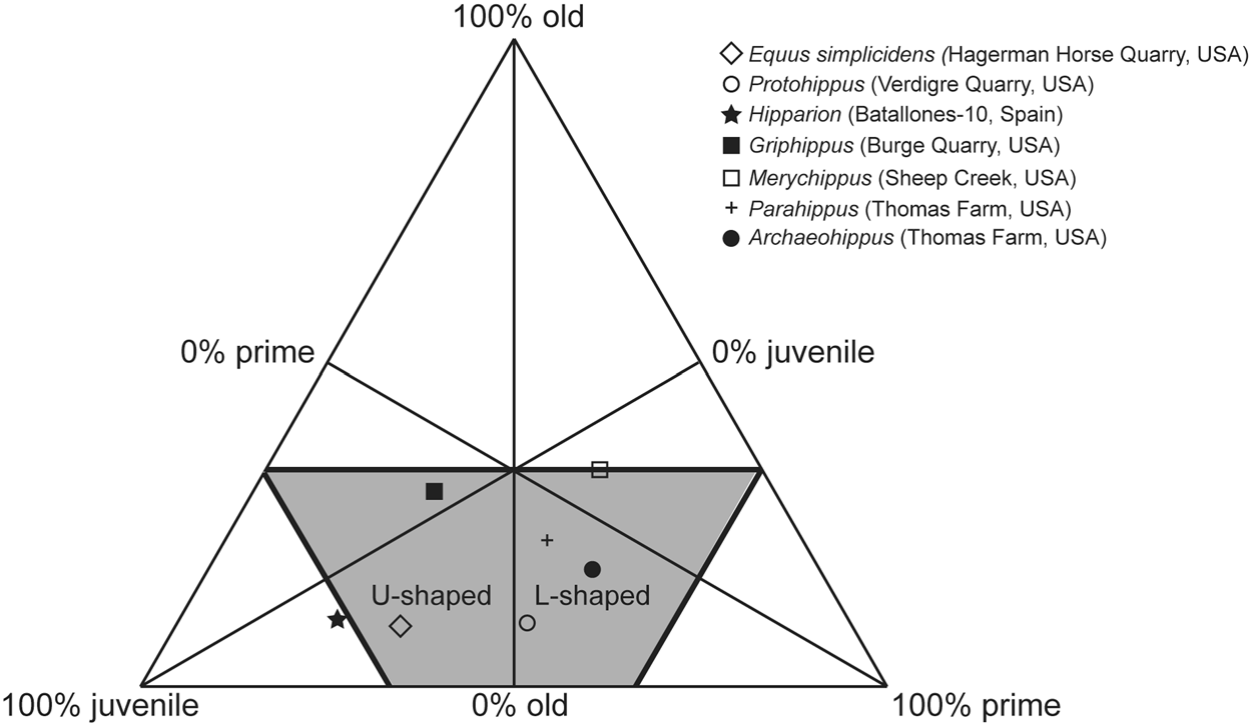
Ternary diagram showing the age distributions of different fossil equid species. See Table 1 and Supplementary Table S3 for further information. The mortality profile of *Griphippus (Pseudohipparion) gratus* from Burge Quarry (Early Pliocene) (not present in Table 1) was described as attritional^22^.

After the analysis of the radiological images, we propose that the order of the permanent cheek teeth mineralization and eruption for hipparionine horses is m1, m2, (p2, p3), p4, m3 (Table 1; Fig. 1; Supplementary Figs S1–S7). Batallones-10 *Hipparion* sp. assemblage is dominated by infantile and juvenile individuals, i.e., they make the 67.9% of the assemblage (Age classes 1, 2 and 3) (Fig. 2). The proportion of juvenile/prime adult/old individuals of the Batallones-10 *Hipparion* sp. projected in a ternary diagram shows this predominance of inmature individuals, although it falls very close to the boundary of the U-shaped (attritional) mortality profile (Fig. 3).

## Discussion

### Dental ontogeny in hipparionine horses and comparison with other extinct and living equids

Dental replacement sequences shed light on the postnatal pace of growth of a species. In this sense, Smith^15^ coined the term ‘Schultz’s rule’, based on the work of the anthropologist Adolph Schultz^14^, to advocate that species with fast life history traits (rapid dental development, fast growth, short life spans) have all three molars erupted before any teeth are replaced, whereas species with slow life history traits (slow dental development, slow growth, long life spans) start replacing the deciduous cheek teeth relatively early, at least before the last molar is erupted^14,15,17,18,41^. In species that grow fast, maxillary bones and mandibles can accommodate the third molars relatively early while the deciduous teeth are still functional. In turn, in slow-growing mammals, the delayed growth of the jaw constrains the eruption of molars and, therefore, the replacement teeth erupt when the milk teeth lose their functionality, which happens before there is space in the jaw to accommodate the second and third molars^41^.

In Table 1, we provide the data on cheek teeth eruption sequences available in the literature for both ancient and modern equid species. We acknowledge that the species listed in Table 1 represent only a meagre representation of all the species known in the very diverse Equidae phylogenetic tree but they still can provide some useful information about the pattern of tooth eruption through time in horses. The species analysed are included in the Anchitheriinae and Equinae subfamilies (there is no information in the literature about dental eruption sequences of the basal Hyracotheriinae subfamily). The Anchitheriinae subfamily is represented by *Parahippus leonensis* and *Archaeohippus blackbergi* (Table 1). The Equinae subfamily is represented by the primitive *Merychippus primus*, the Hipparionini species *Hipparion* sp. (from this study), *Hipparion chiai* and *Hipparion* cf. *H. coelophyes* and the Equini species *Equus simplicidens, Equus zebra, Equus burchelli, Equus hemionus, Equus asinus* and *Equus caballus*. As for *Protohippus* cf*. P. perditus*, some authors include this genus in the Equini tribe^3^ whereas others include it in a separate group (“Protohippini”)^6^.

It can be observed that the pattern of eruption of the p2, p3, m1 and m2 seems to be very conservative through time in the Equidae family (Table 1). All equids here analysed, since the Early Miocene until the present and belonging to different clades, show that the m1 is the first cheek teeth to erupt, followed by the m2. After the m2 eruption, the p2 and p3 erupt almost synchronously (Fig. 1). The p4 and m3 are the last teeth to erupt in horses but, unlike the previously cited permanent cheek teeth, their eruption timing is variable.

Table 1 shows that in equids of the genus *Equus* (starting with *Equus simplicidens*) either the m3 or the p4 can be the last cheek teeth to erupt. Even in individuals belonging to the same *Equus* species, the timing of eruption of p4 and m3 is not fixed (see *Equus zebra* and *Equus burchelli* in Table 1). As for the rest of species, the m3 is always the last cheek teeth to erupt.

In the context of the Schultz’s rule, the fact that the m3 is always the last cheek teeth to erupt in the Anchiteriinae and Hipparionini horses (after the three permanent premolars have erupted) could lead us to think that their jaws grew slower and therefore, the m3 could be accommodated relatively later in time than in modern horses. Smith^15^ compared the eruption sequence with the time of eruption of the M1 in varied mammals as a measure of growth pace. These Anchiteriinae and Hipparionini horses have a dental eruption sequence similar to *Hippopotamus*, which is a very slow-growing mammal whose M1 emerges 2 years after birth. Nevertheless, as indicated before, this very slow growth does not seem to be the true for ancient horses and different authors have underlined that they had shorter life spans and that their ontogenetic dental events and growth were accelerated compared to modern horses^3,19,22–24,34,35^. Rather, the fact that the m3 eruption occurred after the p4 eruption in ancient horses could be related to the fact that the dp4 loses its functionality earlier than in *Equus* horses and before the m3 had enough room to erupt in the mandible. This seems to be true for the Anchitheriinae and the Hipparionini equids regardless their body mass, e.g., *Archaeohippus blackbergi* has an average body mass of 43.9 kg^42^ whereas *Hipparion* sp. from Batallones-10 has an average body mass of 238 kg^43^. The Anchitheriinae species reported in Table 1, that is *Archaeohippus blackbergi* and *Parahippus leonensis*, display another difference in their eruption sequence and is that their p4 erupts at the same time than the p2 and p3^24,26^, which would suggest that the dp4 durability was even shorter than in the other horse species here analysed and it was functional only for the same period of time as the dp2 and the dp3. In turn, in *Equus* horses, the dp4 seems to be functional for a longer time so the mandible has the time to grow until the m3 has space to erupt, which can happen either before or after the p4 erupts.

Given the scarce dental eruption data for the Equidae family, we cannot be sure whether the pattern observed for *Equus* horses is characteristic and limited to this genus or whether it goes back to older Equini representatives. If we regard *Protohippus* cf*. P. perditus* as belonging to the Equini, then we should discard that this pattern is present in all the tribe (in *Protohippus* cf*. P. perditus* the last cheek tooth to erupt is the m3; Table 1).

The timing of p4 and m3 eruption here observed could be related to other traits in the Equidae evolutionary history such as the increase of the teeth crown height (hypsosodonty) and, in view of Table 1, this seems a plausible option. Increased hypsodonty represents an investment in the durability of the dentition in response to (1) increased wear in low-quality habitats and (2) increased reproductive lifespan and longevity^44,45^. These two explanations for increased hypsodonty are not mutually exclusive and, in fact, apply well to the Equidae family^3,4,19,22–24,34,35^. A further test for the hypothesis of a link between the timing of p4 and m3 eruption and hypsodonty could come from the analysis of the eruption sequences of the most hypsodont Hipparionini species such as *Eurygnathohippus*^46^ or *Plesiohipparion*^47^. If these species displayed the pattern described for *Equus*, then we could corroborate the existence of this link but if they rather showed the pattern described for the Anchitheriinae and the Hipparionini (i.e., the m3 is the last tooth to erupt), then it would be plausible to think that the unordered eruption of the p4 and m3 is a trait only present in the *Equus* genus (and maybe some other Equini species not analysed here).

Although the Schultz’s rule seems to be violated in terms of the m3 emergence in horses (i.e., in ancient and relatively faster growing horses the m3 erupts in the last place), if the rule is considered in general terms, the fact that deciduous cheek teeth start being replaced before the last molar erupts indicates that equids ranging from the Early Miocene until the present (Table 1), and including anchitheriine, hipparionine and equine horses, can be considered as slow-growing, long-living mammals. We have not found studies dealing with the dental ontogenetic sequences of the first representatives of the Equidae family (e.g., *Eohippus*, *Orohippus, Epihippus*) to track the Schultz’s rule in the origins of this family. Smith^15^ suggested that the dental pattern where the eruption of all three molars occurs before the replacement of any teeth is considered primitive as it occurred in early mammals, which were small and rapidly growing. We include in Table 1 the study of Hellmund^28^ who described the cheek teeth eruption sequence of the Middle Eocene palaeothere *Propalaeotherium hassiacum* from Geiseltal (Germany). Palaeotheres are a primitive group of perissodactyls that was once considered as true equids but that are currently recognized as belonging to the family Palaeotheriidae that, together with the Equidae, is included in the superfamily Equoidea^3,48^. *Propalaeotherium hassiacum* shows a dental sequence where the three molars erupt before the replacement of the deciduous premolar occurs (Table 1), so after the Schultz’s rule, this primitive horse-related species would be a fast-growing, short-lived taxon. It remains to corroborate whether the same would be true for ancient true horses and, if it is true, when in the horse evolutionary history, the change from fast-growing to slow-growing species would have taken place.

As for the cheek teeth mineralization sequence in the specific case of the hipparionine horses, the radiological analysis of the Batallones-10 *Hipparion* sp. jaws demonstrates that it coincides with the observations made in modern horses. The m1 begins its mineralization most probably while *in uterus* as its germ is clearly visible in very young individuals (Fig. 1a). The m2 is the following cheek teeth to mineralize (Fig. 1b), followed by p2 and p3 whose germs become visible almost synchronously (Supplementary Fig. S2). As for the mineralization timing of the p4 and m3, we observe that the p4 starts to mineralize before the m3 (Fig. 1d), which coincides with the pattern observed in modern Equidae species^39,40^.

When inferring past environmental conditions and feeding behaviour based on the dental enamel of hipparionine horses, stable isotope paleoecologists sample preferentially the m3 (followed by the p4), since it is the least probable cheek tooth to contain the mother’s milk signal^49,50^. This preference of the m3 (and the p4) was based on the study of the cheek teeth mineralization timing of modern equids; hipparionine horses have been assumed to have the same mineralization order than modern horses. Our study, corroborates this assumption for the first time with the direct observations performed on the excellent *Hipparion* sp. fossil material from Batallones-10.

### Taphonomic interpretation of the *Hipparion* sp. mortality profile from Batallones-10

In Fig. 3, we present the juvenile/prime adult/old proportions of the *Hipparion* sp. individuals from Batallones-10 together with data from other fossil horse assemblages.

The mortality type suggested for each of these assemblages is provided in Table 1. The Late Pliocene Hagerman Horse Quarry *Equus simplicidens* assemblage displays a juvenile/prime adult/old proportion very close to the one found at Batallones-10. McDonald^51^ proposed that this assemblage was the result of a catastrophic event based on the wide range of discrete-age classes represented, nevertheless, its position in the ternary diagram and previous interpretations by Gidley^52^ and Gazin^53^ seem to point to an attritional origin for this taphocoenosis. In the case of Batallones-10 *Hipparion* sp. assemblage, although it presents a somehow unique pattern lying outside the U-shaped and L-shaped profile areas, we propose that it was the result of an attritional, gradual mortality. The infant and young *Hipparion* sp. individuals, represented abundantly at Batallones-10, had higher natural risk of mortality than prime and middle-aged adults. *Hipparion* sp. is an ancient equid species and comparisons with modern equids must be made with caution, but a closer view to the infantile and juvenile age classes allows us to broadly assign age class 1 and age class 2 to foals and yearlings, respectively. Horses in these two age classes maintain a very strong bond with their mothers and depend on their milk (above all age class 1 individuals) and accompany them^54^. In modern equids, age class 3 would correspond to the period of time when juveniles disperse from their maternal band, which usually takes place in feral populations when individuals are 2 to 3 years old^54^.

**Figure 4:**
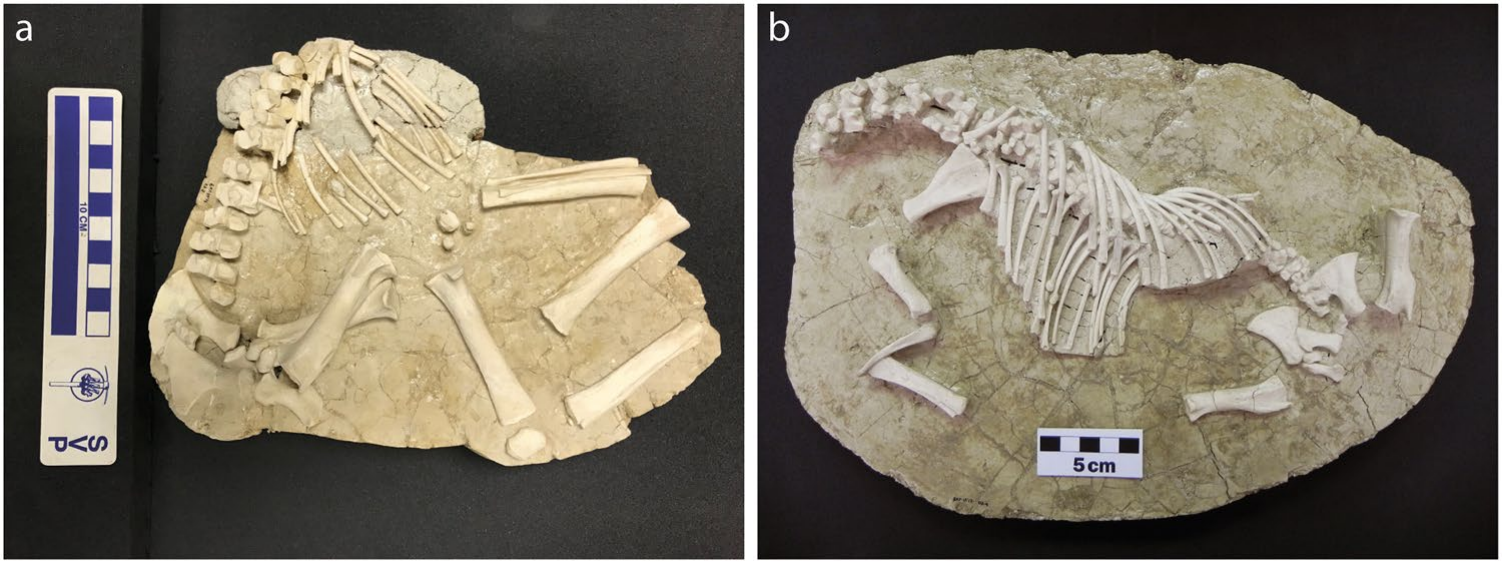
*Hipparion* sp. foetus remains from Batallones-10. (**a**) Foetus discovered in the 2016 field season (BAT-10′16 E3-2). (**b**) Foetus discovered in the 2017 field season (BAT-10′17 D3-9).

**Figure 5:**
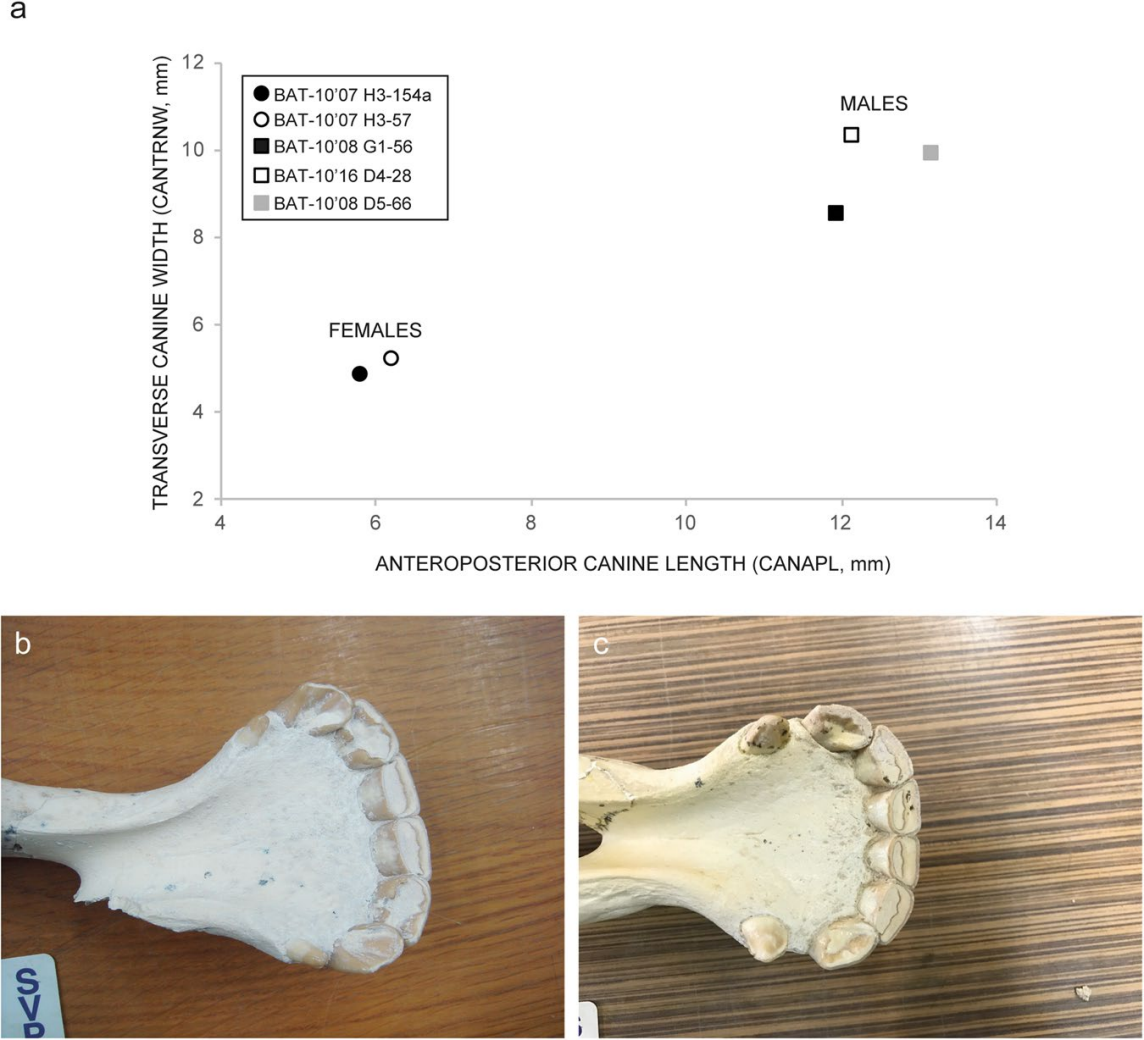
Sexual dimorphism in Batallones-10 *Hipparion* sp. as revealed by canine dimensions. (**a**) Bivariate plot of canine length and width. Measurements as in MacFadden^67^. (**b**) Female individual BAT-10′07 H3-154a. (**c**) Male individual BAT-10′08 G1-56.

In the theoretical attritional model, old individuals should be more abundant than they are in the Batallones-10 assemblage; nevertheless, among the adult individuals we have detected another group that is vulnerable to routine ecologically related deaths. This group is constituted by pregnant females. We have found two *Hipparion* sp. foetus skeletons at Batallones-10 (Fig. 4). Female horses more usually give birth to a single foal^26^ so at least two pregnant females should be accounted for in the Batallones-10 assemblage. Based on measurements of the canines, we have been able to determine the sex of five adult individuals whose mandibles still preserved the anterior dentition. Two mares and three stallions have been identified (Fig. 5). Since much of the *Hipparion* fossil material is disarticulated and dispersed in the Batallones-10 area, it is not possible to know whether the two foetuses belonged to the two mares identified based on their canines.

Stable isotope analyses performed on serially-sampled m3 enamel of several of these individuals (M.S. Domingo, unpublished data) points to the existence of a significant hydrological seasonality in the Madrid Basin at 9.0 Ma. In environments with seasonal availability of resources, births tend to be seasonal^26^. Seasonal breeding produce populations displaying discrete dental age classes, however, this discreteness can only be detected in the fossil record if 1) a catastrophic mortality took place or 2) an attritional mortality of seasonal character occurred^3,19,22,24^. Batallones-10 *Hipparion* sp. mandibles analysed in this study does not exhibit discrete dental age classes; rather they seem to show a continuum of dental events above all in the infant and juvenile stages (Fig. 1; Supplementary Figs S1–S7). Given the attritional character that we suggest for Batallones-10 assemblage, there are two options possible (1) that there was seasonal breeding but attritional death happened year-round, then capturing individuals with no discrete dental age classes or (2) that there was not seasonal breeding (irrespective of the seasonal or non-seasonal character of death).

Batallones-10 sediments were deposited in a small shallow water-hole^55^. The presence of remains of tortoises and frogs also supports the existence of a water body in this area. Death of modern equids around water bodies during droughts have been reported in the literature^56,57^. Joubert^56^ indicated that the heaviest mortality in the Hartmann zebra (*Equus zebra hartmannae*) from Khomas Hochland (Namibia) during a drought period occurred among newly-born, young foals and pregnant females. These individuals died around waterholes or got trapped in the mud of a dam that was drying up^56^. Berger^57^ reported that several foals and a yearling in the Owyhee Desert (Nevada, USA) died when they became trapped in the mud of a water spot after following their mothers to drink. We suggest as a plausible hypothesis to explain the Batallones-10 *Hipparion* sp. assemblage that vulnerable individuals died around a waterhole in the course of one or several drought periods occurring 9 million years ago.

## Conclusions

Complete sequences of dental ontogeny are rare in the fossil record but provide valuable information that otherwise would remain unknown or would be inferred from modern counterparts. Specifically, they shed light on the patterns of mineralization, eruption, and replacement of the teeth and on aspects of the life history of extinct species, as well as on the causes of death of the fauna and mode of formation of fossil sites. We have studied in this paper the cheek teeth ontogeny of hipparionine horses, a key group in the Equidae family during the Late Miocene and Pliocene, and have compared it to other extinct and living horse species. To achieve this purpose, we performed radiological analysis on excellently-preserved mandibles of 28 *Hipparion* sp. individuals, that range from neonatal to mature stages, from the site of Batallones-10 (Cerro de los Batallones Madrid Basin, Spain). This is the first time that the cheek teeth ontogeny of an extinct equid species, including juvenile individuals, is analysed with radiological techniques.

We have defined 7 age classes for Batallones-10 *Hipparion* sp. and have determined that the sequence of mineralization and eruption of hipparionine horses is: m1, m2, (p2, p3), p4, m3. The sequence of mineralization is similar to the sequence observed in modern *Equus* but differences arise regarding the eruption pattern. Specifically, we have observed that in members of the Anchitheriinae subfamily and Hipparionini tribe the m3 always erupts after the p4 while in horses of the *Equus* genus either the p4 or the m3 might erupt in the last place. This might be related to the shorter durability of the deciduous tooth dp4 in the anchitheriine and hipparionine horses (compared to the *Equus* genus) that is shed and replaced by the p4 before the m3 has room in the mandible to emerge. This fact could be linked to the acquisition of a potential longer functionality of cheek teeth throughout the evolutionary history of horses as their hypsodonty increased. From the general viewpoint of the Schultz’s rule, hipparionine horses, like other horses spanning from Early Miocene species until the present *Equus*, are slow-growing, long-living mammals.

Additionally, this study corroborated for the first time in an extinct horse species that m3 and p4 should be the preferred cheek teeth to sample in stable isotope paleoecological analyses, as these are the last cheek teeth to mineralize and provide dietary signals not influenced by the mother’s milk.

Individuals from groups with the highest risk of natural mortality (infants and yearlings dependent on their mothers, as well as pregnant females) are predominant at Batallones-10, so we considered that this accumulation was the result of a gradual, attritional mortality. The death of weak individuals gathered around a water body during drought periods constitutes a plausible hypothesis for the accumulation of hipparionine individuals at Batallones-10.

## Material and Methods

### Material

We performed radiographs and CT scans of hemimandibles and complete mandibles of 28 *Hipparion* sp. individuals from the fossil site of Batallones-10 (Supplementary Table S1) to document the sequence and pattern of mineralization, eruption and replacement of cheek teeth (premolars and molars). We worked with mandibles instead of skulls and maxillary bones for several reasons: (1) mandibles and hemimandibles account for more individuals than skulls and maxillae at Batallones-10, so information obtained from the latter would be redundant, (2) hemimandibles are easily radiographed whereas skulls and upper-jaw bones would have to be analysed with CT scans which are far more time consuming and, (3) at the time of this study, there were more mandibles and hemimandibles prepared than skulls. The fossil material here analysed is housed at the National Museum of Natural Sciences-CSIC (Madrid, Spain).

Like the rest of the mammals, horses replace their teeth only once during their lifetime (diphyodonty) and possess deciduous and permanent dentitions although molars only have one generation^58^. In this study, dp2-dp4 are the lower deciduous teeth, p2–p4 are the lower permanent premolars and m1-m3 are the lower molars. The vestigial p1 (wolf tooth) was absent from all the analysed mandibles.

Individuals here analysed range from neonatal specimens to specimens with advanced degree of wear in their permanent teeth. In hypsodont horses, as is the case of hipparionine horses, only a small portion of the crown is in wear at any given time; crown reserves remain under the gum line and erupt gradually through the lifetime of the animal as the exposed part of the crown wears away. Then, although eruption and replacement patterns can be analysed from the visual inspection of the mandibles, mineralization patterns can only be observed with radiographic imaging (or by dissecting the mandibles). This type of imaging is also an enormous help to estimate the wear stage of the teeth.

### Radiological imaging

Radiographs were performed on hemimandibles whereas CT scans were carried out on complete mandibles (Fig. 1; Supplementary Figs S1–S7). Radiographs were carried out at the Faculty of Veterinary Medicine of the Complutense University of Madrid using a X-ray equipment Sedecal Neovet-V with a digital radiography flat panel detector Perkin Elmer XRPad 4336. The parameters selected for the radiographic procedure were 70 kV and 6.4 mAs. CT scans were performed at the Military Centre of Veterinary (Spanish Ministry of Defence) with a Philips MX 4000 dual CT scan, at 140 kV and 160 mA, 1.2 mm slice thickness, 1.0 mm increments, and an image matrix of 768 × 768.

### Analysis of age classes

We placed the mandibles in successive age classes based on the presence of tooth crypts and germs, eruption and replacement patterns, and wear degree. Wear degree was based on the qualitative visual inspection of the height of the teeth in the radiological images. We consider that a tooth starts erupting when it emerges through the mandible and complete eruption is reached when the tooth reaches the height of the rest of the teeth in the mandible.

Based on data coming from demographic studies of modern mammalian communities, two theoretical types of mortality profiles have been described: (1) attritional profile (=U-shaped profile) and (2) catastrophic profile (=L-shaped profile)^59–61^. In the attritional pattern young and, to a lesser extent, old individuals are overrepresented relative to their live abundances^62^. In the catastrophic profile, also known as ‘living-structure pattern’ because of its resemblance to the instantaneous age structure of a live population^61,62^, successively older age classes are represented by fewer and fewer individuals, i.e., it corresponds to the age profile of a typical stable population^62^. Attritional mortality results from normal or routine ecologically related deaths (disease, malnutrition or accidents) of naturally weaker population members. In turn, catastrophic/living-structure profile can be caused by catastrophic events, such as floods and volcanic eruptions, but also by non-catastrophic events such as, for example, accidental falls into cavities or ambush strategy predation where, in principle, there is no prey selection^61,62^. The age-frequency distributions of Batallones-10 *Hipparion* sp., along with those of other fossil Equidae species with age-frequency distributions available in the literature, are represented in the ternary diagram proposed by Stiner^61^ with the aim of obtaining a simplified overview of the fossil assemblage age distribution and untangling the cause of death (Fig. 3; Supplementary Table S3). The corners of the ternary plot represent 100% juvenile individuals (individuals with some of the deciduous dentition still in place), 100% prime adult individuals (individuals with all the deciduous teeth shed and the permanent dentition in place) and 100% old individuals (individuals with the permanent dentition heavily worn and some teeth showing more than half of the crown worn away).

### Data availability

All data generated or analysed during this study are included in this published article (and its Supplementary Information files).

## Electronic supplementary material

Supplementary Figures S1-S7

Supplementary Tables S1-S3
