# Supplementary material for: First Radiological Study of a Complete Dental Ontogeny Sequence of an Extinct Equid: Implications for Equidae Life History and Taphonomy
Source: Sci Rep. 2018 May 31;8:8507. doi: 10.1038/s41598-018-26817-3 (PMC5981301; doi:10.1038/s41598-018-26817-3)
Supplement: Supplementary file 1 — Supplementary Figures S1-S7 [file 41598_2018_26817_MOESM1_ESM.pdf]

Supplementary Figures for:

FIRST RADIOLOGICAL STUDY OF A COMPLETE DENTAL ONTOGENY SEQUENCE OF AN EXTINCT EQUID: IMPLICATIONS FOR EQUIDAE LIFE HISTORY AND TAPHONOMY

M. Soledad Domingo, Enrique Cantero, Isabel García-Real, Manuel J. Chamorro Sancho, David M. Martín Perea, M. Teresa Alberdi, Jorge Morales

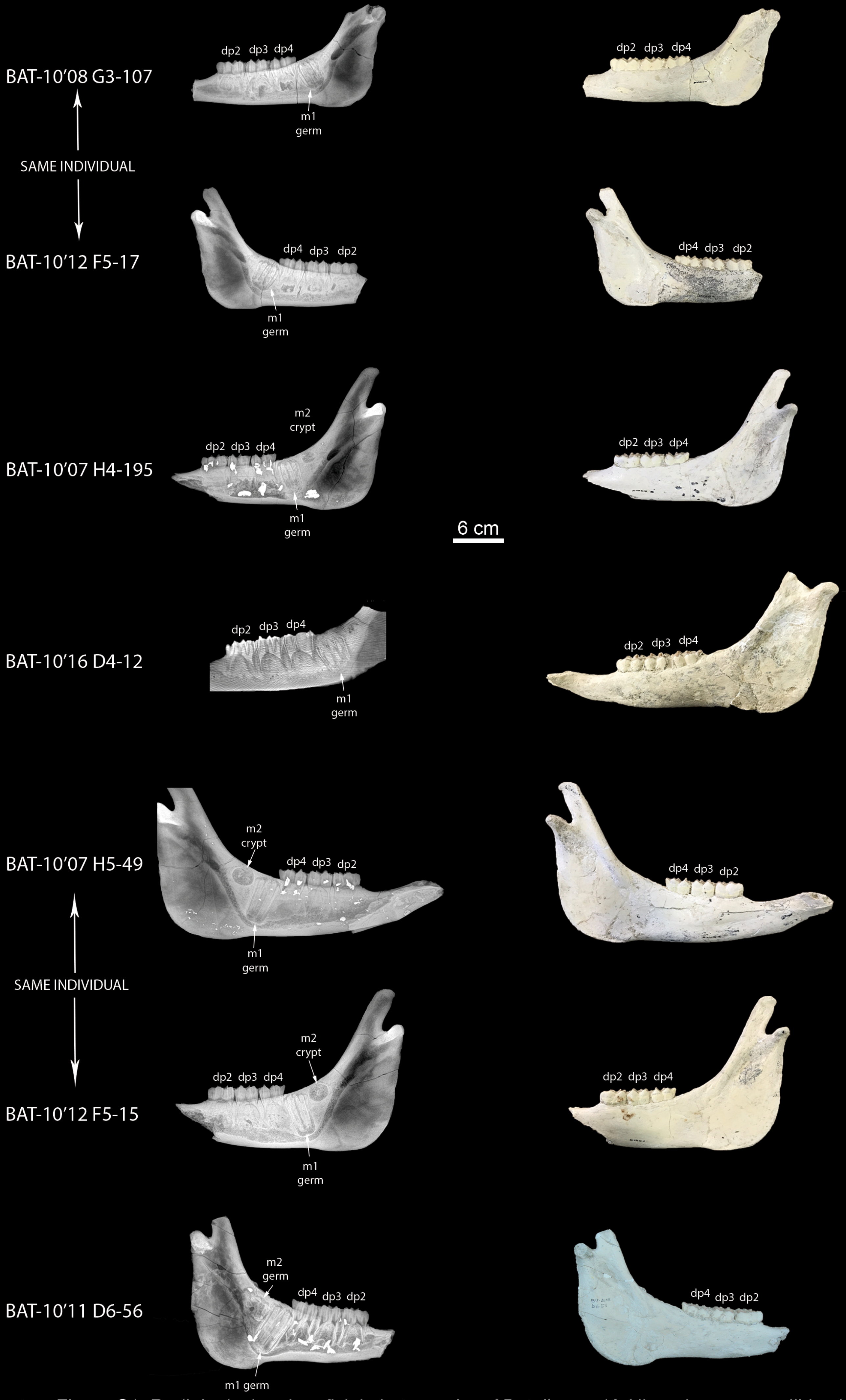

Supplementary Figure S1. Radiological and surficial photographs of Batallones-10 *Hipparion* sp. mandibles included in the Age Class 1.

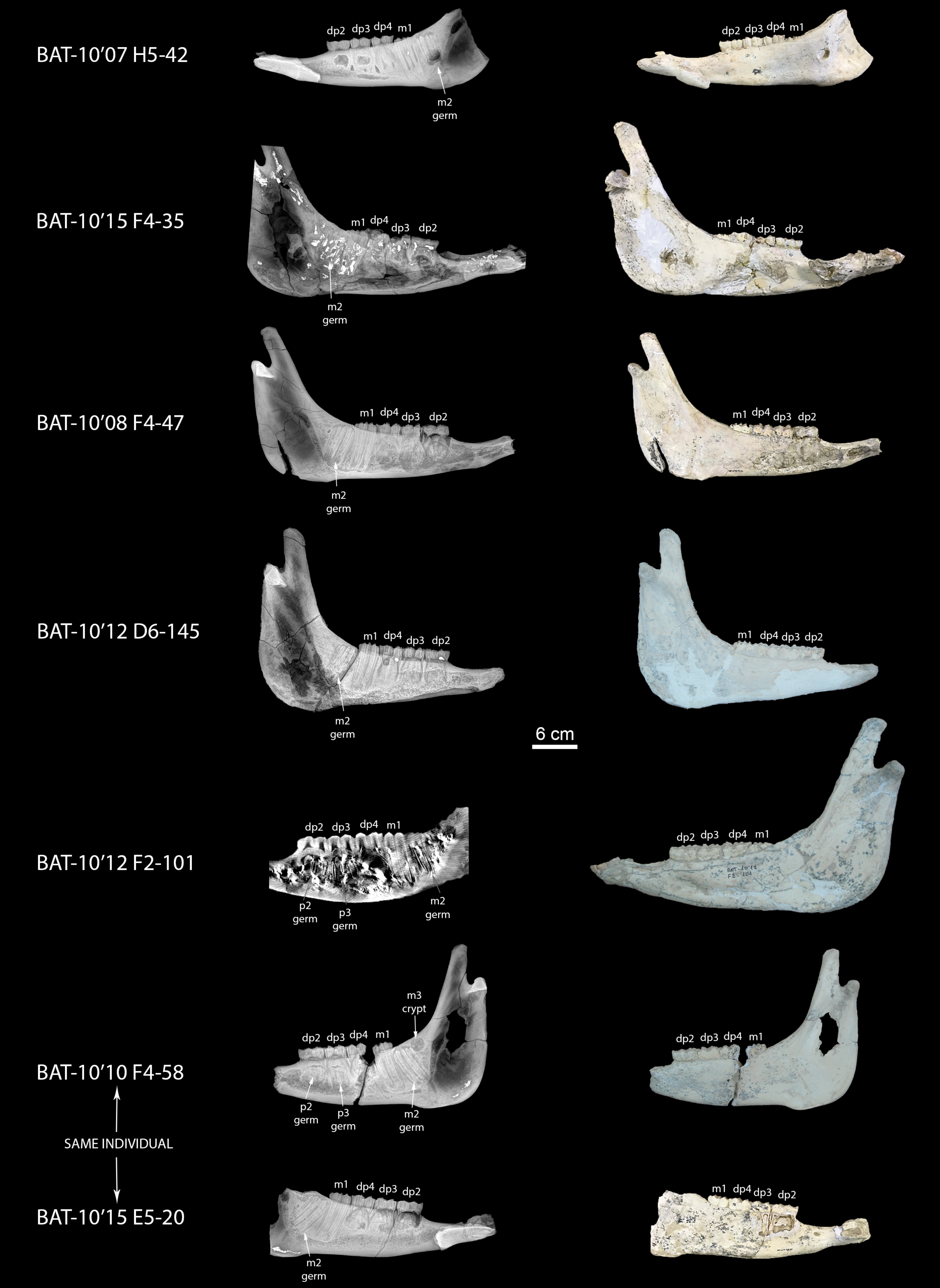

Supplementary Figure S2. Radiological and surficial photographs of Batallones-10 *Hipparion* sp. mandibles included in the Age Class 2.

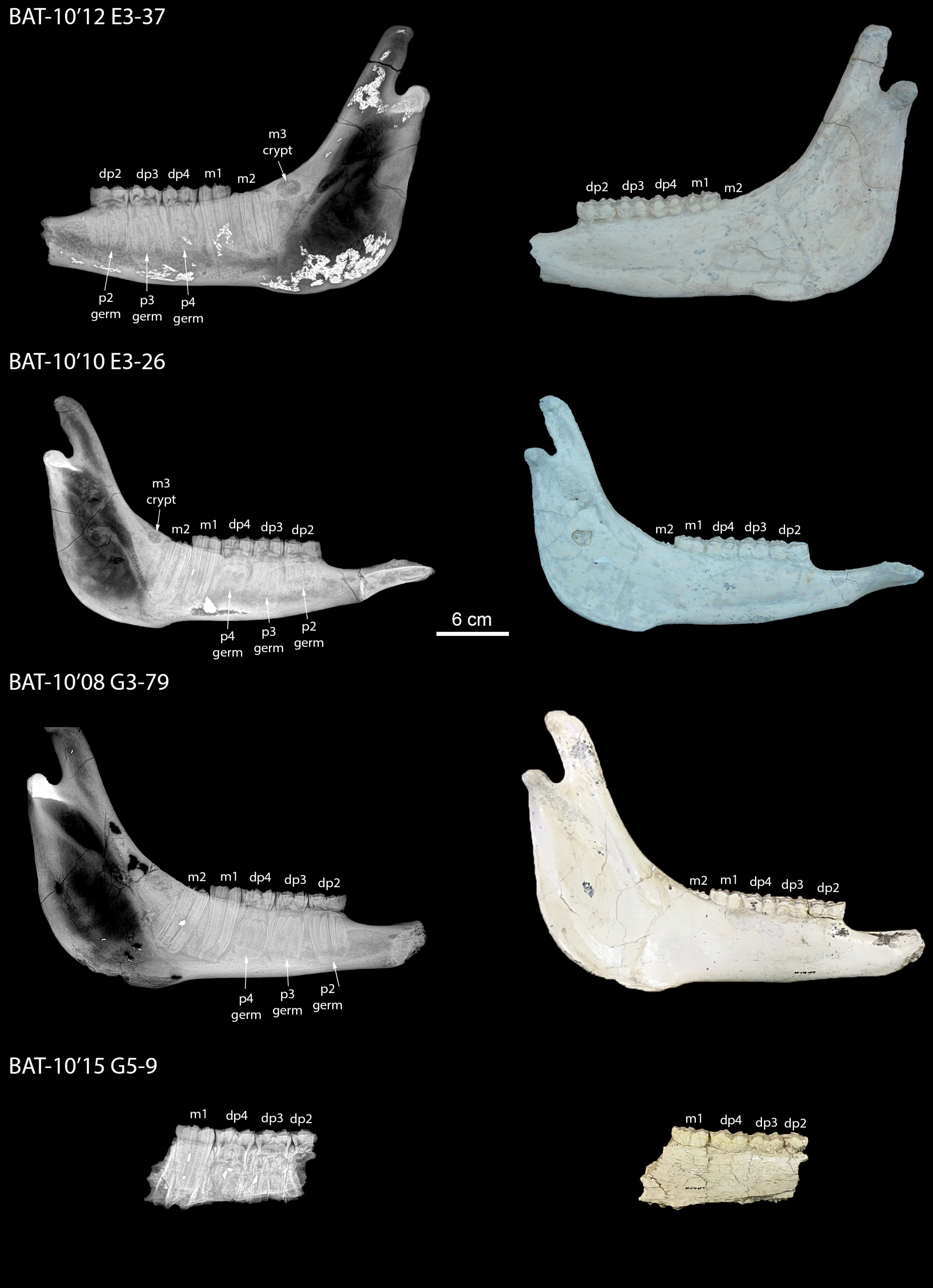

Supplementary Figure S2. *Continuation.*

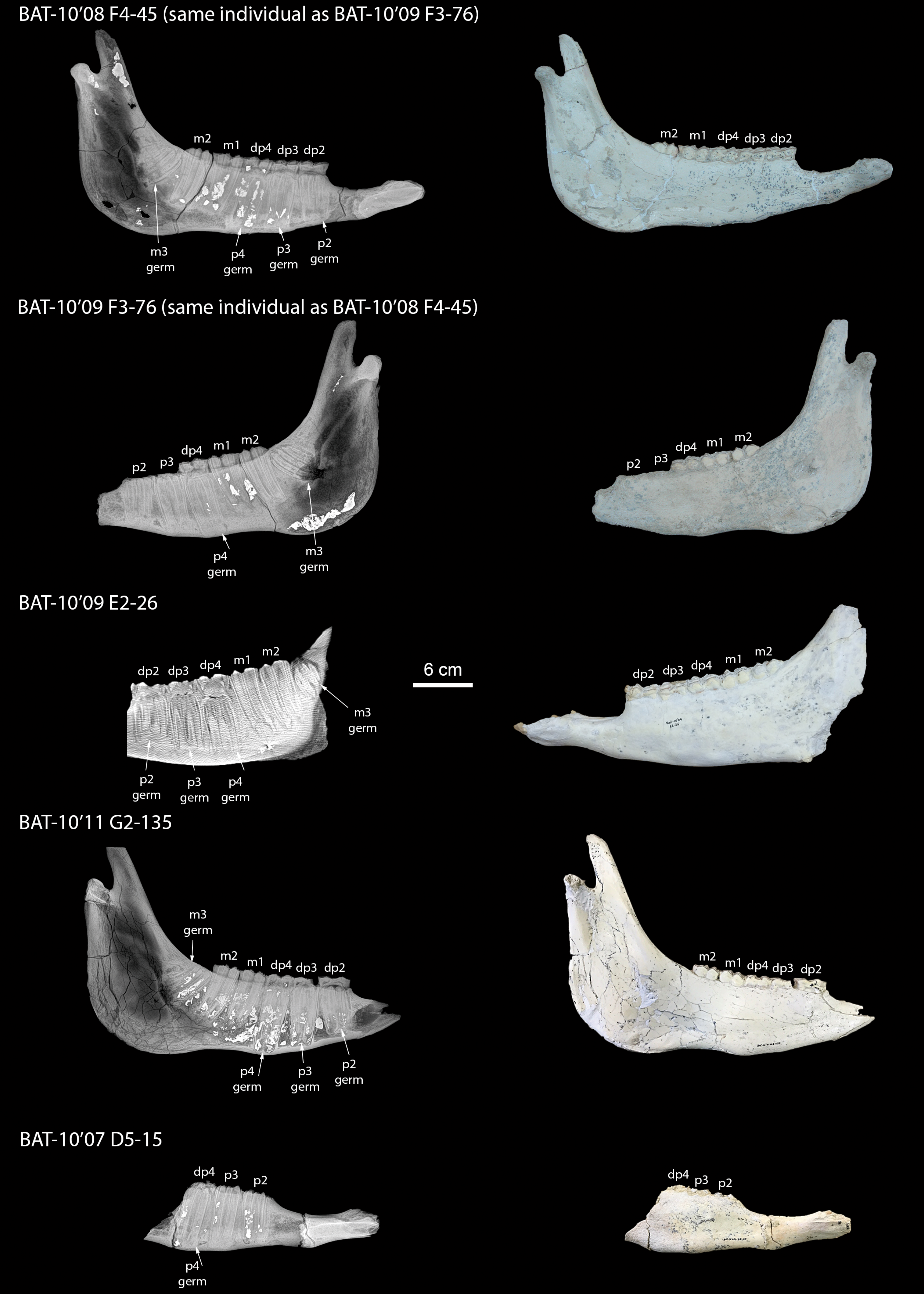

Supplementary Figure S3. Radiological and surficial photographs of Batallones-10 *Hipparion* sp. mandibles included in the Age Class 3.

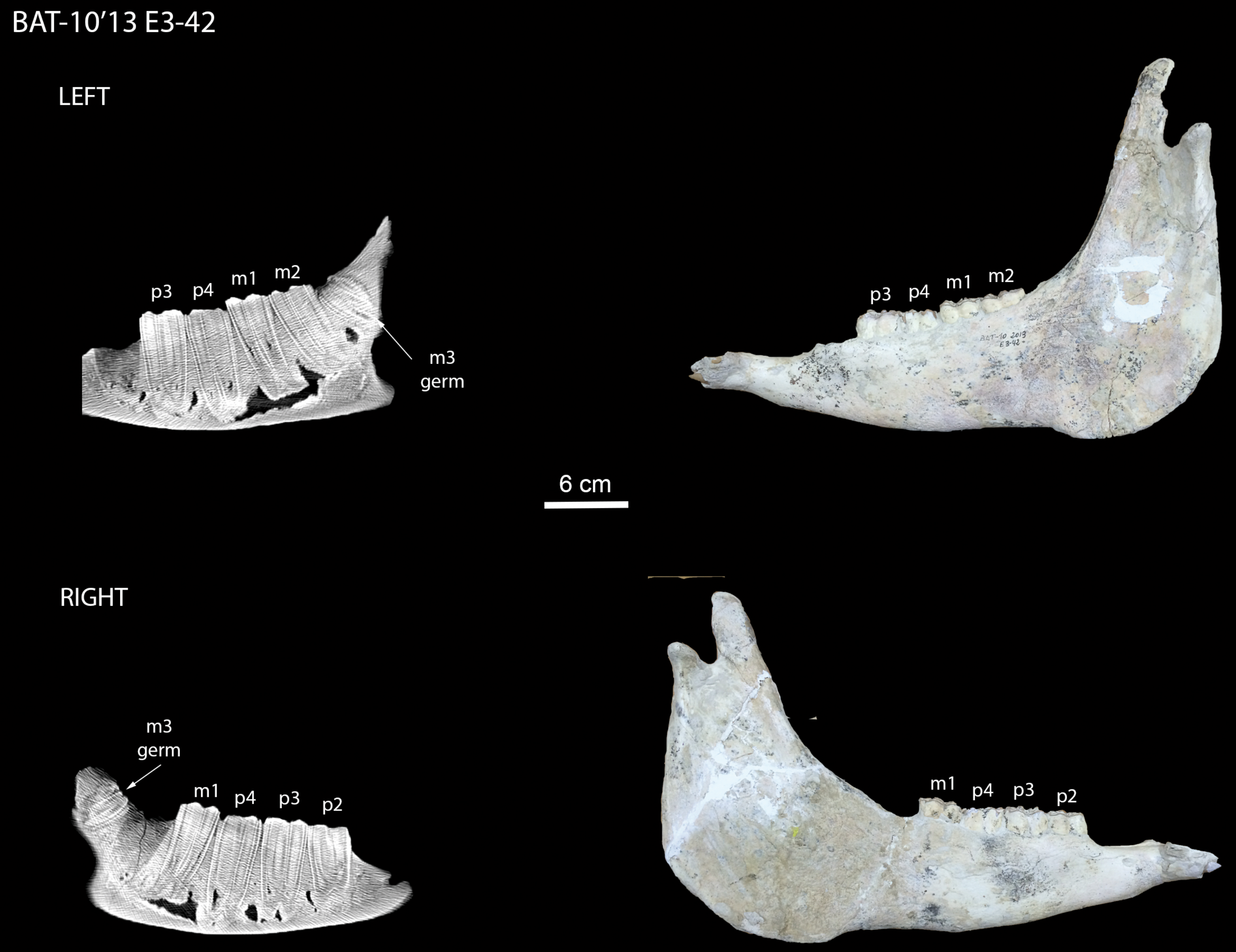

Supplementary Figure S4. Radiological and surficial photographs of Batallones-10 *Hipparion* sp. mandibles included in the Age Class 4.

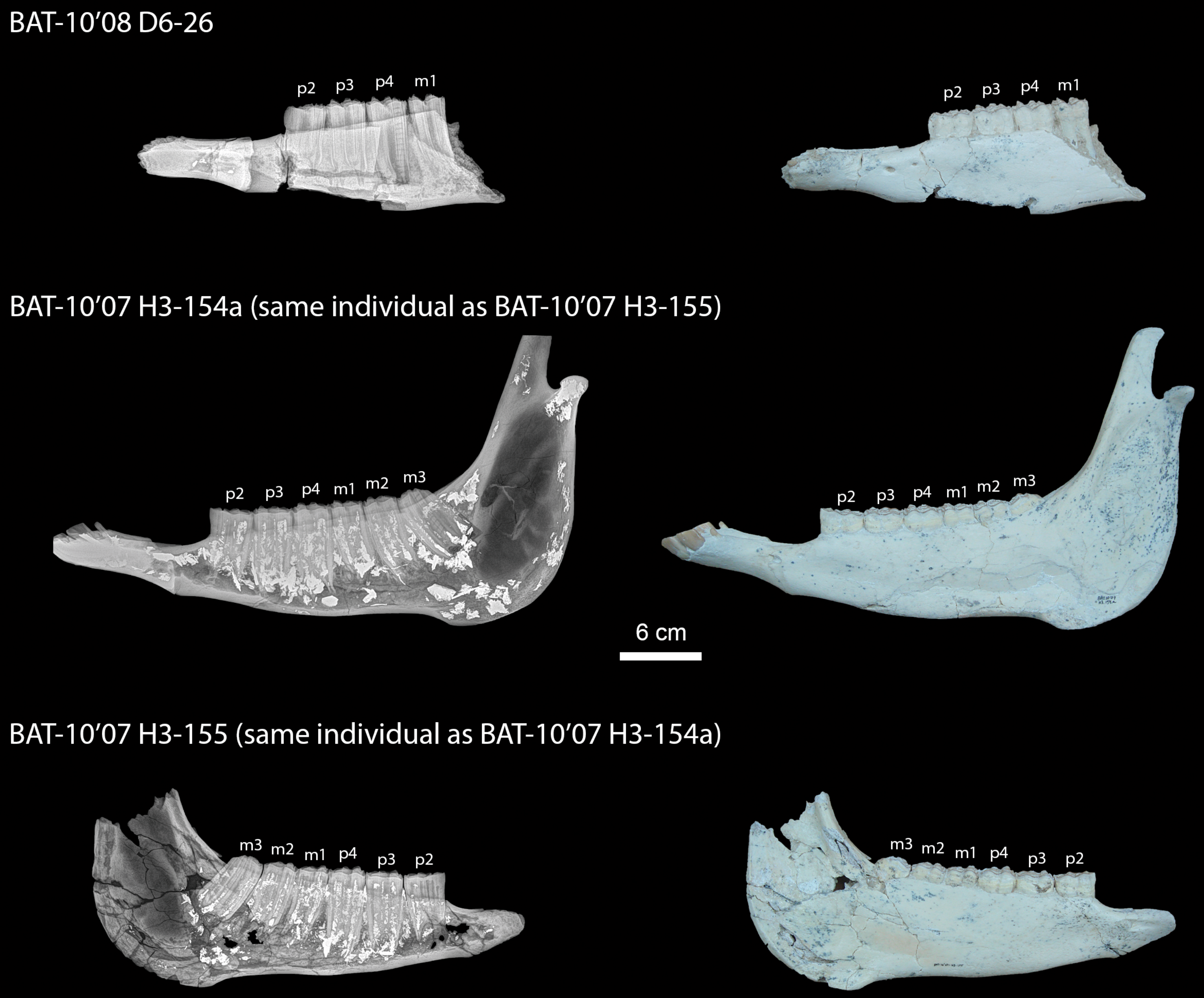

Supplementary Figure S5. Radiological and surficial photographs of Batallones-10 *Hipparion* sp. mandibles included in the Age Class 5.

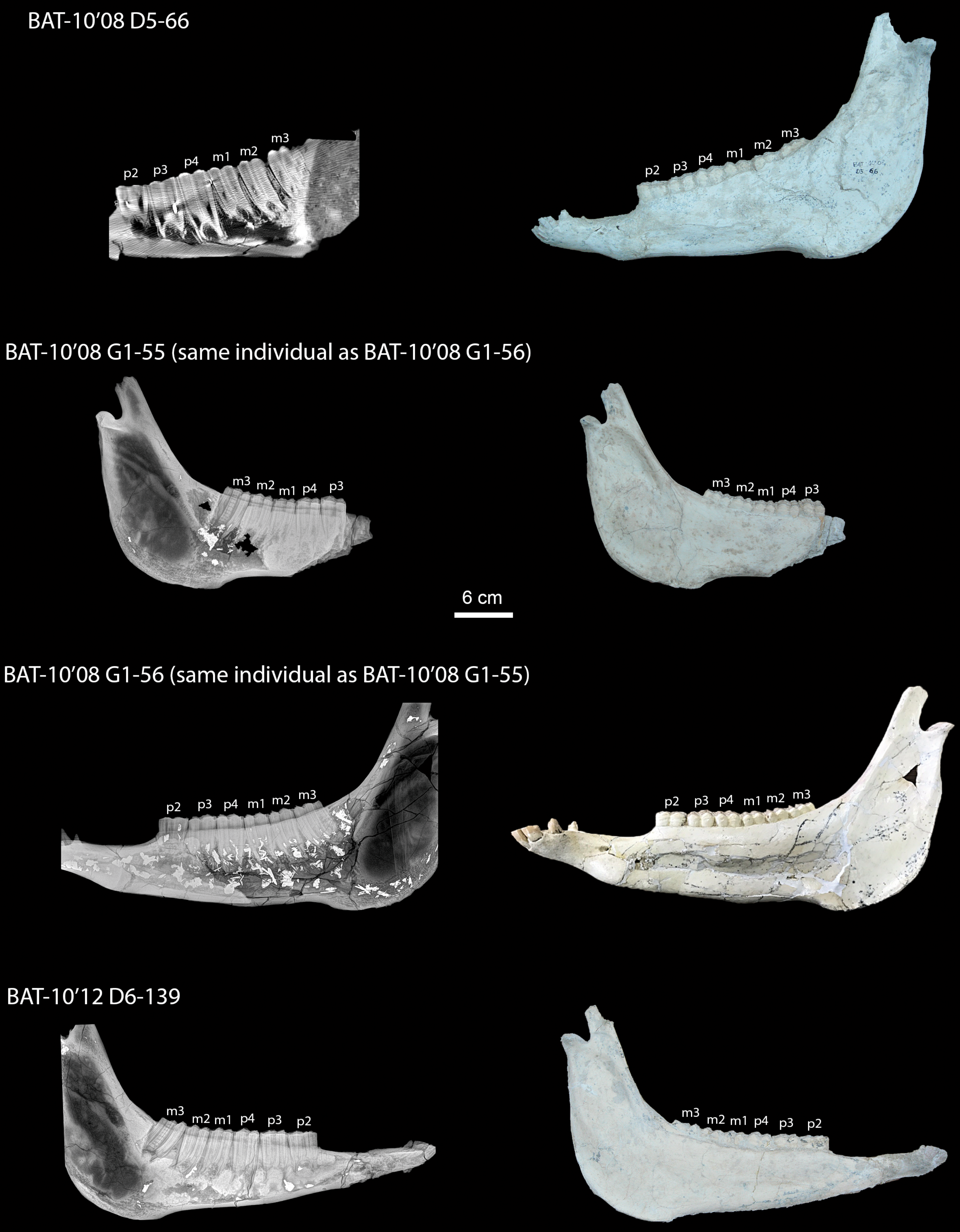

Supplementary Figure S6. Radiological and surficial photographs of Batallones-10 *Hipparion* sp. mandibles included in the Age Class 6.

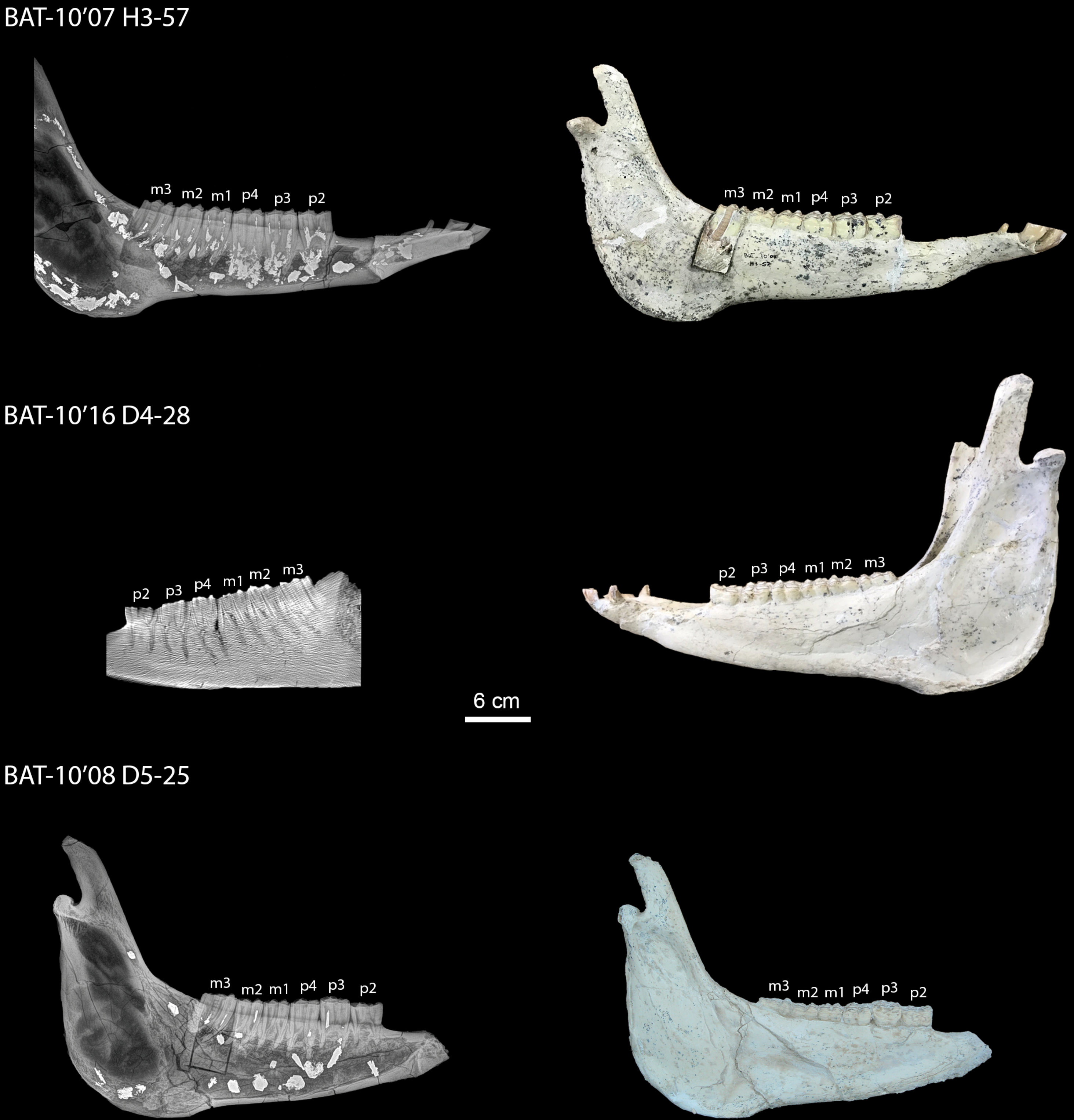

Supplementary Figure S7. Radiological and surficial photographs of Batallones-10 *Hipparion* sp. mandibles included in the Age Class 7.
